# Serum Parathyroid Hormone Responses to Vitamin D Supplementation in Overweight/Obese Adults: A Systematic Review and Meta-Analysis of Randomized Clinical Trials

**DOI:** 10.3390/nu9030241

**Published:** 2017-03-06

**Authors:** Ashley Lotito, Masaru Teramoto, May Cheung, Kendra Becker, Deeptha Sukumar

**Affiliations:** 1 Department of Nutrition Sciences, College of Nursing and Health Professions, Drexel University, Philadelphia, PA 19102, USA; arlotito@gmail.com (A.L.); mmc372@drexel.edu (M.C.); kendravos@gmail.com (K.B.); 2 Division of Physical Medicine & Rehabilitation, School of Medicine, University of Utah, Salt Lake City, UT 84108, USA; Masaru.Teramoto@hsc.utah.edu

**Keywords:** parathyroid hormone, 25 hydroxy vitamin D, obesity, overweight, vitamin D supplementation

## Abstract

Obesity is often associated with vitamin D deficiency and secondary hyperparathyroidism. Vitamin D supplementation typically leads to the reductions in serum parathyroid hormone (PTH) levels, as shown in normal weight individuals. Meanwhile, the dose of vitamin D supplementation for the suppression of PTH may differ in overweight and obese adults. We conducted a systematic review and meta-analysis of randomized controlled trials to determine the dose of vitamin D supplementation required to suppress PTH levels in overweight/obese individuals. We identified 18 studies that examined overweight or obese healthy adults who were supplemented with varying doses of vitamin D3. The primary outcomes examined were changes in PTH and serum 25-hydroxyvitamin D (25OHD) levels from baseline to post-treatment. The results of the meta-analysis showed that there was a significant treatment effect of vitamin D supplementation on PTH, total standardized mean difference (SMD) (random effects) = −0.38 (95% CI = −0.56 to −0.20), *t* = −4.08, *p* < 0.001. A significant treatment effect of vitamin D supplementation was also found on 25OHD, total SMD (random effects) = 2.27 (95% CI = 1.48 to 3.06) *t* = 5.62, *p* < 0.001. Data from available clinical trials that supplemented adults with D3 ranging from 400 IU to 5714 IU, showed that 1000 IU of vitamin D supplementation best suppressed serum PTH levels, total SMD = −0.58, while vitamin D supplementation with 4000 IU showed the greatest increase in serum 25OH levels. Vitamin D and calcium supplementation of 700 IU and 500 mg, respectively, also showed a significant treatment effect on the suppression of PTH with a total SMD = −5.30 (95% CI = −9.72 to −0.88). In conclusion, the meta analysis of available clinical trials indicates that 1000 IU vitamin D supplementation can suppress serum PTH levels, while 4000 IU of vitamin D was associated with the largest increase in serum 25OHD levels in the overweight and obese population.

## 1. Introduction

Low vitamin D status is common in the United States, with higher rates of deficiency reported among those with greater adiposity [1,2]. In the 2011 dietary reference intakes (DRIs) report, the Institute of Medicine (IOM) defines vitamin D deficiency as a serum 25-hydroxyvitamin D (25OHD) concentration of less than 12 ng/mL, and inadequacy as a serum 25OHD of 12–20 ng/mL [1] and these limits of normalcy have been challenged by other professional organizations [3]. Vitamin D deficiency is associated with many of the same health risks as obesity, such as cardiovascular disease, certain cancers, hypertension, osteoporosis, and secondary hyperparathyroidism [2,3,4,5]. 

The synthesis and secretion of parathyroid hormone (PTH) is higher in those with vitamin D deficiency [4,6]. Both PTH and 25OHD play important roles in calcium homeostasis [6]. In the kidney, PTH triggers the hydroxylation of 25OHD to its active form, 1α,25-dihydroxy-vitamin D (1α,25(OH)2D), which enhances the intestinal absorption of calcium [4]. Chronic elevations of serum PTH increase osteoclast activity and the urinary excretion of phosphorous, thus having a negative impact on bone density [4,6]. Low dietary calcium, skeletal muscle wasting, primary or secondary hyperthyroidism, chronic kidney disease (CKD), or inadequate vitamin D status may be independent contributors to high serum PTH concentrations [4].

Alterations in the vitamin D endocrine system often occur with obesity [7,8,9,10,11]. Increased adiposity is reported to be positively associated with serum PTH levels and inversely associated with serum 25OHD levels [7,8,9]. The current explanation for this phenomenon is the increased sequestration of 25OHD in excess subcutaneous fat, ultimately decreasing the bioavailability of vitamin D for calcium absorption [7,10,11]. The diminished availability of serum 25OHD causes a compensatory increase in PTH secretion to maintain serum calcium concentrations. 

Calcium supplementation is associated with suppression of serum PTH; however, recent data suggest a significant increase in the risk of myocardial infarction and stroke with calcium supplementation [12]. This consequence on cardiovascular health may be due to the up-regulation of calcium-sensing receptors on the parathyroid gland, thus facilitating vascular calcification and impairing function of vascular cells [12]. Vitamin D supplementation can also suppress serum PTH concentrations, yet overweight and obese individuals may require a higher dose of vitamin D to obtain the same effect [13,14,15]. Vitamin D supplementation in combination with adequate dietary calcium improves serum 25OHD concentrations [13,14], and the absorption of calcium, subsequently suppressing PTH secretion without increasing the risk of cardiovascular complications. 

The recommended daily allowance (RDA) of vitamin D is 600 IU/day for adults [1]. To reach an adequate vitamin D status of over 20 ng/mL, overweight and obese individuals may need higher doses [5]. Moslehi et al. recently published a meta-analysis of studies investigating the PTH response to vitamin D supplementation in adult populations, reporting that a vitamin D dose of 3000 IU/day for one year was effective in the suppression of PTH [15]. Currently, there is limited research regarding the dose response to vitamin D on PTH levels in those with excess adiposity. This systematic review and meta-analysis aims to quantitatively determine the lowest dose of vitamin D supplementation required to significantly reduce PTH levels in the overweight and obese population. 

## 2. Methods

### 2.1. Inclusion and Exclusion Criteria

We included original clinical research articles in this meta-analysis. Specific study inclusion criteria were: study reported dosages of vitamin D supplementation, serum PTH or 25OHD levels at baseline and end of the study, and examined overweight and/or obese populations with a body mass index (BMI) of 25 kg/m^2^ and above who were healthy adults, 18 years of age or older. Studies examining participants who were adolescents or children, had normal BMI, including a weight loss intervention, patients with renal disease and other metabolic alterations, or had undergone gastric bypass or bariatric surgery were excluded from this meta-analysis. If a study reported PTH/25OHD values but did not have a control group, it was also excluded from the meta-analysis.

### 2.2. Search Strategy

**Figure 1:**
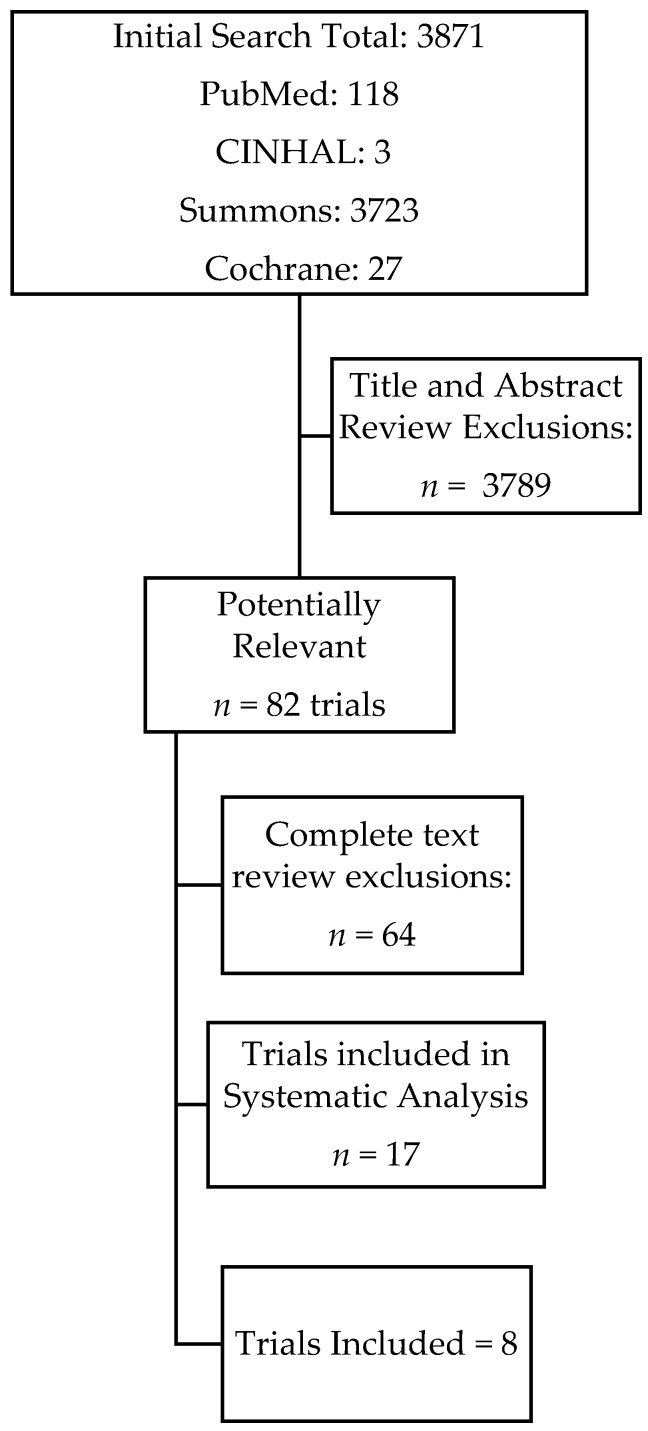
Flowchart for the process of identification of trials.

Literature searches were completed using PubMed, CINHAL, Summons, and Cochrane databases. We conducted database searches and utilized both medical subject headings and free text search terms. Date restrictions were not used, but the search was limited to studies published in English with human participants. The results were narrowed down using the following key terms: “vitamin D supplementation and parathyroid hormone and obesity”, “vitamin D supplementation and parathyroid hormone and obese”, and “vitamin D supplementation and parathyroid hormone and overweight”. Figure 1 demonstrates the flow of the trials selected.

### 2.3. Data Collection

One author (AL) screened the titles and abstracts identified in the above search strategy. The full texts of potentially relevant studies were retrieved, and the inclusion and exclusion criteria were applied. The following data were extracted from each study, as summarized in Table 1: the number of participants in the study (sample size), mean BMI, duration of the study, vitamin D supplementation dose, and calcium supplementation dose if applicable, baseline and end values of serum 25OHD and PTH levels, and change in PTH and serum 25OHD during the intervention. PTH levels and 25OHD levels are reported as pg/mL and ng/mL, respectively. The duration of the studies are reported in weeks. Vitamin D supplementation dose were converted to IU/day if the supplementation was given weekly or monthly.

### 2.4. Data Extraction and Analysis

The primary outcome variables of interest were the changes in PTH and 25OHD before and after the intervention. The interventions that included a vitamin D supplementation and vitamin D combined with calcium supplementation were analyzed. The study is in the forest plot analysis if: (1) it measured the outcome variable(s) before and after the intervention; and (2) it reported the change in mean and standard deviation of the differences in the outcome variable(s) before and after the intervention. Although several studies reported initial and final change in outcome variables, only those that reported changes (With Mean and SD) before and after intervention were included in the forest plot analysis.

A separate meta-analysis was performed on the data of: (1) PTH responses by vitamin D supplementation; (2) PTH responses by vitamin D combined with calcium supplementation; (3) 25OHD responses by vitamin D supplementation; and (4) 25OHD responses by vitamin D combined with calcium supplementation. We used MedCalc (Ver. 16.2.0; MedCalc Software bvba, Ostend, Belgium) for the meta-analysis to calculate standardized mean difference (SMD) using Hedges g [34], with a 95% confidence interval (CI) as an effect size for each study, and Cochran’s Q and I2 statistics for heterogeneity across the studies. SMD was tested with an α level of 0.05, whereas, an α level of 0.10 was used to examine Cochran’s Q, as suggested by Higgins et al. [35]. In terms of determining the total treatment effect, we used a fixed effects model when there was a non-significant Cochran’s Q, while a random effects model was used in the case of a significant Cochran’s Q (i.e., presence of heterogeneity). Forest plots for each of the four meta-analyses above were also produced to illustrate the treatment effects for each study as well as the overall treatment effect across the studies. In addition, funnel plots were produced and inspected to examine publication bias [36,37] if there were 10 or more studies included in the meta-analyses [38,39]. Lastly, a sensitivity analysis was conducted using Stata (Ver. 14.2; StataCorp., LLC, College Station, TX, USA) to assess the between-study heterogeneity and the relative influence of an individual study on the overall effect size. We used the leave-one-out approach in which the overall effect is recalculated after a study is excluded one by one [40,41]. Cohen’s criteria are used to determine effect size for this analysis [42].

## 3. Results 

### 3.1. Characteristics of Clinical Trials

As seen in Figure 1, 3871 publications were retrieved from the 4four databases searched. After the initial screening of the titles and abstracts of the studies, the full texts of 82 publications were retrieved and analyzed against the inclusion and exclusion criteria, resulting in the exclusion of 64 publications. There were no further publications identified from screening references lists of these 82 full-text publications. As a result, a total of 18 publications were included in this report, with 11 of them being included in the meta-analysis of PTH responses by vitamin D supplementation, three included in the analysis of PTH responses by vitamin D and calcium supplementation, and the other four being analyzed qualitatively. Table 1 [16,17,18,19,20,21,22,23,24,25,26,27,28,29,30,31,32,33] shows the publications included in this report in the order they were retrieved. The table lists the sample size, mean BMI, mean change in PTH and 25OHD, as well as baseline and end of study results for PTH and 25OHD. The sample sizes varied from *n* = 23 to 441, averaging at 152 participants per study. The studies included males and females, males only, or females only. We excluded studies that involved a weight loss intervention. This is because weight loss independently raises serum 25OHD levels. 

### 3.2. PTH and 25OHD Responses by Vitamin D Supplementation

**Figure 2:**
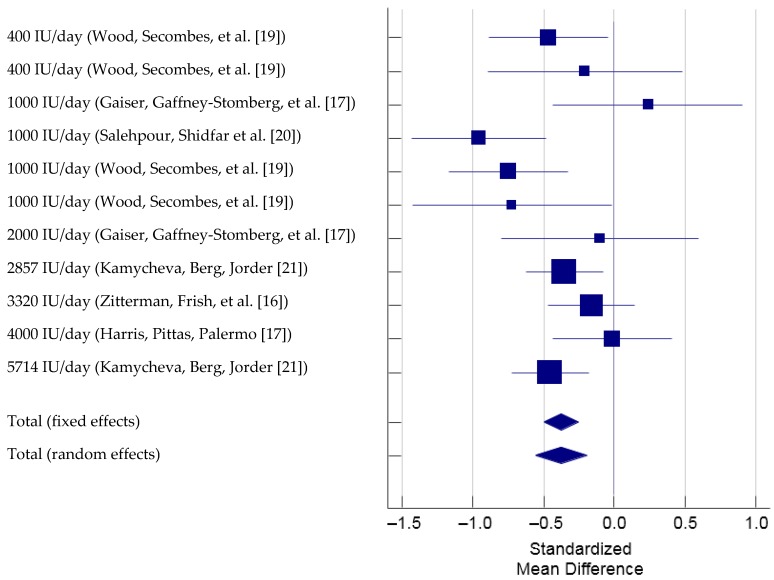
Forest plot of Standardized Mean Difference of PTH with vitamin D supplementation. Note: Study by Wood et al., 2014 examined overweight and obese populations separately and is listed as two separate analysis in this Forest plot.

There were six studies that used 11 different vitamin D supplementation amounts or participants (i.e., overweight and obese participants separately), which met the inclusion criteria and therefore were included in the meta-analysis. (total *N* = 1077, Figure 2) [16,17,18,19,20,21]. We reported the changes in 25OHD observed in these studies and did not interpret whether these levels reached adequacy or not according to IOM or the Endocrine society guidelines.

**Figure 3:**
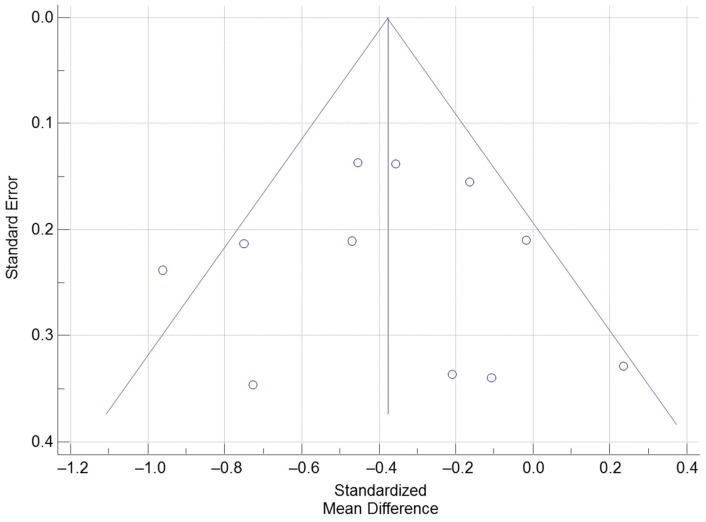
Funnel Plot examining trials of PTH responses with vitamin D supplementation.

The Cochran’s Q was significant according to the criteria suggested by Higgins et al. (2003) [35], Q(10) = 19.70, *p* = 0.032. The *I*^2^ was 49.2% (95% CI = 0.0% to 74.6%), indicating that 49.2% of the total variation across the studies could be due to heterogeneity. Overall, there was a significant treatment effect of vitamin D supplementation on PTH, total SMD (random effects) = −0.38 (95% CI = −0.56 to −0.20), small effect size based on the Cohen’s criteria (1988) [42], *t* = −4.08, *p* < 0.001. Based on the inspection of the forest plot, it appeared that vitamin D supplementation with a dose of 1000 IU produced the greatest reduction in PTH. The total SMD of the reduction in PTH by 1000 IU of vitamin D supplementation calculated from the three studies that used 1000 IU of vitamin D supplementation was −0.58 (95% CI = −1.06 to −0.11) [17,19,20]. The funnel plot was roughly symmetrical (Figure 3) suggesting that publication bias was unlikely. According to the sensitivity analysis, the estimated SMDs ranged from −0.42 to −0.33, with all SMDs being significantly different from 0, which indicated that the significant overall treatment effect was not determined by a single trial.

**Figure 4:**
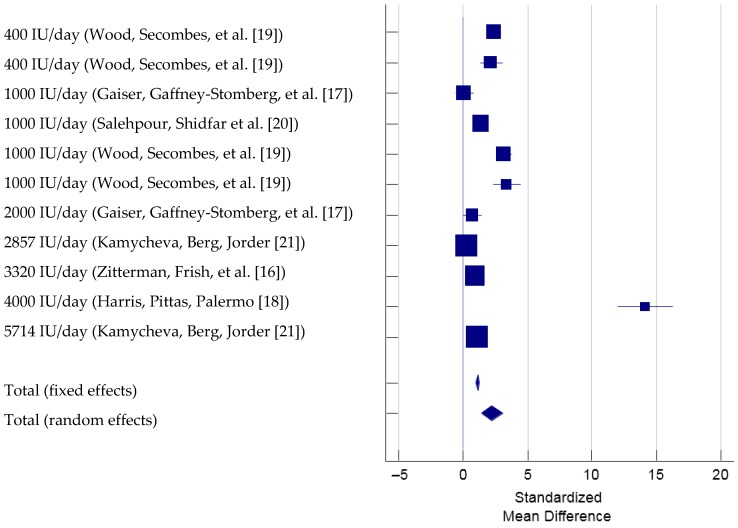
Forest Plot showing Standardized Mean Difference of 25OHD with vitamin D supplementation. Note: Study by Wood et al., 2014 examined overweight and obese populations separately and is listed as two separate analysis in this Forest plot.

**Figure 5:**
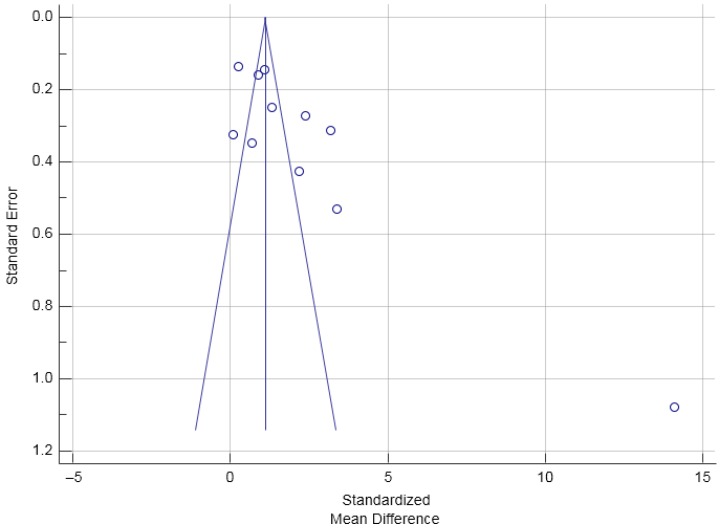
Funnel Plot examining trials of 25OHD responses with vitamin D supplementation.

To determine the effect of vitamin D supplementation on the changes in 25OHD, there were six studies that used 11 different vitamin D supplementation, met the inclusion criteria and therefore were included in the meta-analysis (total *N* = 1077, Figure 4) [16,17,18,19,20,21]. The Cochran’s Q was significant, Q(10) = 286.59, *p* < 0.001. The *I*^2^ was 96.5% (95% CI = 95.1% to 97.5%), indicating that 96.5% of the total variation across the studies could be due to heterogeneity. Overall, there was a significant treatment effect of vitamin D supplementation on 25OHD, total SMD (random effects) = 2.27 (95% CI = 1.48 to 3.06), small effect size based on the Cohen’s criteria (1988), *t *= 5.62, *p* < 0.001. Based on the inspection of the forest plot, it appeared that vitamin D supplementation with a dose ranging from 400 IU to 5714 IU induced similar increases in 25OHD except that one study using 4000 IU of vitamin D caused a substantially larger effect, SMD = 14.13 (95% CI = 11.98 to 16.27) [18]. The funnel plot (Figure 5) showed one outlying trial by Harris et al. (2012) reporting a markedly larger intervention effect (i.e., larger SMD) compared with other trials with a lower study precision (i.e., larger standard error). Per the sensitivity analysis, when the trial by Harris et al. (2012) was excluded, the estimated SMD was 1.528 and significantly different from 0 (95% CI = 0.94 to 2.12). When any one of the other trials was excluded, the estimated SMDs ranged from 2.16 to 2.54, with all SMDs being again significantly different from 0. Hence, the significant overall treatment effect was not due to any single trial. 

### 3.3. PTH and 25OHD Responses by Vitamin D Combined with Calcium Supplementation

**Figure 6:**
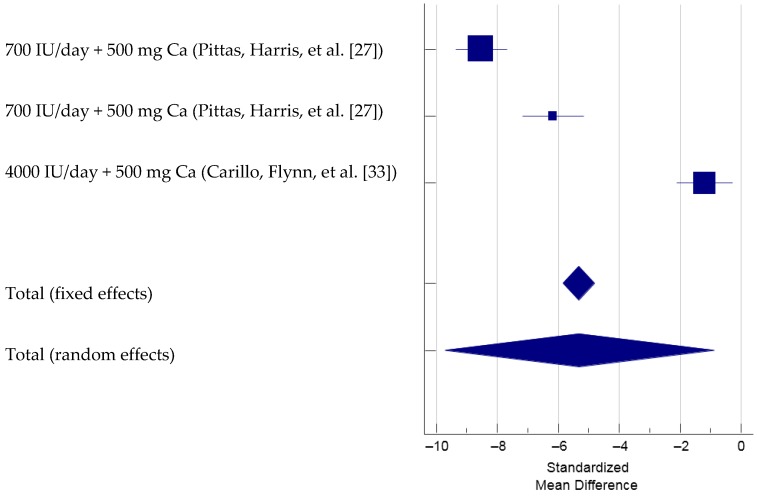
Forest plot showing Standardized Mean Difference of PTH with vitamin D and calcium supplementation. Note: Pittas et al., 2007 study included two separate study groups, once with impaired fasting glucose and another with normal fasting glucose [27].

For determining the responses of PTH due to combined vitamin D-calcium supplementation, two studies that examined three different groups of overweight and obese participants that met the inclusion criteria were included in the meta-analysis (total *N* = 337, Figure 6) [27,33]. The Cochran’s Q was significant, Q(2) = 146.59, *p* < 0.001. The *I*^2^ was 98.6% (95% CI = 97.6% to 99.2%), indicating that 98.6% of the total variation across the studies could be due to heterogeneity. Overall, there was a significant treatment effect of vitamin D combined with calcium supplementation on PTH, total SMD (random effects) = −5.30 (95% CI = −9.72 to −0.88), medium effect size based on the Cohen’s criteria (1988), *t* = −2.36, *p* = 0.019. Based on the inspection of the forest plot, it appeared that vitamin D supplementation with a dose of 700 IU [27], when combined with calcium, produced greater reduction in PTH than did 4000 IU of vitamin D with calcium [33]. Since there were only three trials included in this meta-analysis, the funnel plot was not produced and inspected [37,38]. Two of the four meta-analyses in this manuscript only contain data from three studies. Hence, the funnel plots for this small number of studies are not useful. As a result, there are only two funnel plots examined in this manuscript. From the sensitivity analysis, the estimated SMDs ranged from −7.41 to −3.73, with all SMDs being significantly different from 0, indicating that the overall treatment effect was not determined by a single trial.

**Figure 7:**
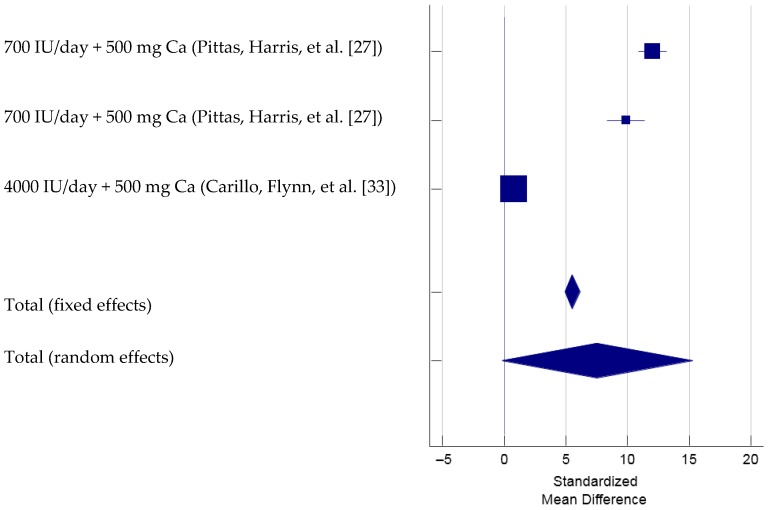
Forest Plot of Standardized Mean Difference of 25OHD with vitamin D and calcium supplementation. Note: Pittas et al., 2007 study included two separate study groups, once with impaired fasting glucose and another with normal fasting glucose [27].

To determine the 25OHD responses by vitamin D combined with calcium supplementation, there were two studies with three different doses or populations that met the inclusion criteria and therefore was included in the meta-analysis (total *N* = 337, Figure 7) [27,33]. The Cochran’s Q was significant, Q(2) = 281.16, *p* < 0.001. The *I*^2^ was 99.3% (95% CI = 98.9 to 99.5), indicating that 99.3% of the total variation across the studies could be due to heterogeneity. Overall, there was not a significant treatment effect of vitamin D combined with calcium supplementation on 25OHD, total SMD (random effects) = 7.56 (95% CI = −0.17 to 15.30), *t* = 1.92, *p* = 0.055. Based on inspection of the forest plot, it appeared that vitamin D with a dose of 700 mg combined with calcium supplementation yielded larger treatment effect on 25OHD (SMD = 12.06 and 9.86, respectively) [27] than 4000 IU of vitamin D combined with calcium whose treatment effect on serum 25OHD levels was minimal (SMD = 0.81) [33]. Since there were only three trials included in this meta-analysis, the funnel plot was not produced and inspected [20,21]. The sensitivity analysis showed that the overall treatment effect was significant when the trial by Carillo et al. (2013) [33] was excluded (SMD = 11.08, 95% CI = 8.96–13.19). On the other hand, when any one of the other two trails was excluded, the estimated SMD was not significantly different from 0 (i.e., 95% CI of SMD overlapped 0). 

**Figure 8:**
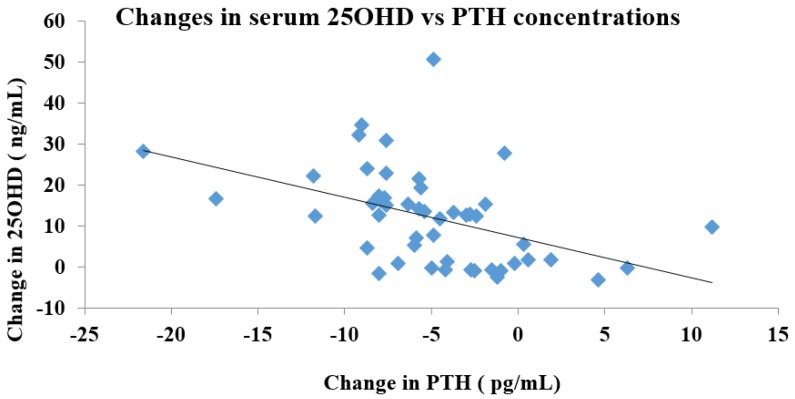
Correlation analysis of changes in 25OHD concentrations vs. changes in PTH concentrations. Pearson’s correlation coefficient is reported as *r* value.

Furthermore, a correlation analysis was performed to determine the relationship between the changes in PTH and 25OHD concentrations (Figure 8). An increase in serum 25OHD levels was associated with a greater suppression of PTH levels (*r* = −0.455, *p* < 0.01).

## 4. Discussion 

Elevated concentrations of serum PTH are associated with numerous adverse health outcomes and commonly coincide with obesity [4,6]. Vitamin D supplementation is used as a clinical treatment for hyperparathyroidism in addition to treating vitamin D deficiency caused by a multiple diseases and medications, although the dose of vitamin D required to suppress PTH in overweight-obese individuals is not yet determined. Our meta-analysis demonstrated that 1000 IU/day of vitamin D was associated with a small, yet statistically significant reduction in serum PTH concentrations. Whether or not this reduction in serum PTH levels is clinically meaningful is not entirely clear. Furthermore, vitamin D supplementation within the range of 400 to 5714 IU per day, on average, was associated with a statistically significant increase of serum 25OHD levels in overweight and obese populations with the greatest increase found with 4000 IU of vitamin D. Our findings indicate an association between the pairing of 700 IU of vitamin D with 500 mg of calcium and a statistically significant reduction (medium effect size) in PTH; however, serum 25OHD concentrations did not significantly increase. Moslehi et al. in their meta-analysis of examining calcium and vitamin D supplementations reported the suppression of PTH (−8.0 pg/mL mean pooled difference) with vitamin D supplementation and a significant PTH suppression (−22.48 pg/mL) in overweight and obese participants with calcium supplementation of 600–1200 mg/day for 12 months [15]. Although our meta-analysis reveals a small effect size, our data do support a true effect of 1000 IU of vitamin D supplementation in the suppression of PTH. In all of the reviewed studies, the control groups showed no significant changes in PTH when compared to their corresponding treatment groups [16,19,25,26,27,28]. In addition, supplementing with active vitamin D leads to significant increases in circulating serum 25OHD concentrations, reducing the need to synthesize PTH thereby successfully suppressing it.

Although mixed, there is evidence linking elevated serum PTH and lower 25OHD to a variety of negative health outcomes in both men and women [43,44,45,46,47,48,49,50,51,52]. Some researchers hypothesize excess PTH secretion to be involved in the development of hypertension, a theory supported by data indicating a positive correlation between blood pressure and serum concentrations of PTH [53,54,55]. In a cross-sectional study of 1250 postmenopausal women, bone loss and fractures were associated with elevated serum PTH and vitamin D insufficiency, particularly in those with a higher BMI [55,56]. This negative impact on bone health could be due to the increased excretion of phosphorous associated with high levels of PTH [55]. Furthermore, an elevated PTH is associated with an increased risk of cardiovascular morbidity and mortality [57,58,59].

Evidence has shown an increased incidence of cardiovascular disease with PTH levels in the upper quartile of normal range, demonstrating that even slight elevations in serum PTH can affect cardiovascular health [58,59]. Elevated PTH has been associated with increased aortic pulse pressure and impaired endothelial function [60], which could partially explain the relationship between PTH and cardiovascular disease.

Vitamin D metabolism is altered in obesity, evidenced by low serum 25OHD levels. The proposed mechanisms include a lack of sun exposure, modified vitamin D activation, and increased vitamin D storage in adipose tissue [7,43,61]. More specifically, one hypothesis states that obese individuals may avoid solar ultraviolet radiation thus diminishing the cutaneous synthesis of vitamin D_3_ from 7-dehydrocholesterol [61]. Alternatively, the activation of 25OHD to 1α,25(OH)_2_D may be enhanced in obese populations, which causes the negative feedback of hepatic production of 25OHD and subsequent lower serum concentrations. More recent research hypothesizes that the deposition of vitamin D_3_ in subcutaneous fat increases with adiposity, subsequently decreasing the bioavailability of vitamin D_3_ as well as circulating serum 25OHD levels [7]. There is a graded decrease in circulating concentrations of serum 25OHD as the BMI increases with the highest incidence of deficiency in the morbidly obese population [44]. Understanding the mechanism(s) behind vitamin D metabolism in obesity may help individualize the treatment of vitamin D deficiency and PTH suppression. The health outcomes of low serum 25OHD in the obese population are also poorly understood. In several large community based cohort studies, low serum 25OHD levels have not shown to be associated with poor CVD outcomes [45,47]. The relationship of low serum 25OHD levels and insulin resistance and metabolic syndrome has been more consistently reported [48,49], although prospective studies do not always suggest improvements in insulin sensitivity with improvements in vitamin D status [50,51]. Whether or not the dosages used in these studies and effective in raising serum 25OHD levels is often debated. Furthermore, the amount of vitamin D supplementation needed to raise serum levels in obese adults has been shown to be different than that of normal weight adults. In normal weight adults, for every 1000 IU of vitamin D supplementation, serum 25OHD has been shown to increase by 4.8 to 20 ng/mL [5,11,14,15]. Out of the few studies investigating vitamin D status in obese populations, some researchers reported that participants receiving 1000 IU/day of vitamin D experienced an increase in serum 25OHD of approximately 10–15 ng/mL [15,18,19,24], while others reported similar or slightly greater increases in 25OHD at 2000–3000 IU/day [20,22,23]. This meta-analysis shows that a range of 400 to 5714 IU of vitamin D sufficiently increases serum 25OHD in obese populations, with 4000 IU showing the largest increase in serum levels of 25OHD.

The relationship between the serum levels of 25OHD and maximal suppression of PTH has been assessed in a few studies. Results from one investigation show that serum 25OHD needs to be higher than 31 ng/mL to suppress PTH in normal weight adult populations [57]. For obese populations, maximal PTH suppression is observed when 25OHD is at 11.1 ng/mL, while the overall population sees maximal PTH suppression when 25OHD reaches 21.7 ng/mL in the overall population [57]. In obese African American women, maximal PTH suppression is achieved at 16.8 ng/mL serum 25OHD [62]. Similar results are recounted in older adults with an average BMI of 26.7 kg/m^2^, and maximal suppression of PTH was seen at 16–20 ng/mL [63]. However, this observation is not consistent and some studies do not demonstrate a threshold at all [64]. These studies suggest differences in physiological response may play a role in the variation of serum 25OHD concentration needed for maximal suppression of PTH, which helps to explain why obese individuals require a higher dose of vitamin D to sufficiently lower their PTH levels. Both age and gender also influence the PTH suppression of 25OHD. In a report of 33,055 women and 77,118 men available in an abstract form, it was observed that sex and age influenced the level at which 25OHD caused the PTH levels to plateau. In men aged 21–30 years, PTH levels plateaued when 25OHD was 35–39 ng/mL whereas in females of the same age range, the plateau was reached when 25(OH)D was 30–34 ng/mL This difference was more dramatic in men aged 41–50 years in whom the PTH levels plateaued when the 25(OH)D reached 30–34 ng/mL whereas in females of the same age range, the PTH levels plateaued at 20–24 ng/mL [65,66]. Our analysis did not statistically examine the maximal suppression of PTH by 25OHD levels, although we observed a strong negative correlation between changes in PTH levels with increasing 25OHD concentrations. However, our analysis also suggests that, 4000 IU vitamin D supplementation produced a substantially large increase in serum 25OHD levels, suggesting that a lower dose of vitamin D supplementation may be able to physiologically suppress serum PTH levels and the suppression may plateau beyond doses higher than 1000 IU despite increases in serum 25OHD levels.

To the best of our knowledge, this is the first systematic review of the effect of vitamin D supplementation on the suppression of PTH in the overweight- obese population. All studies included were randomized controlled trials in overweight and obese populations, and reported mean changes in PTH and 25OHD. Meanwhile, the results of our analysis were unable to distinguish the dose response of overweight versus obese populations or males versus females, as the outcome measurements by these classification variables were not always reported. In addition, the inclusion and exclusion criteria for the meta-analysis limited the number of studies to be included and analyzed, making it difficult to generalize the results of the meta-analysis. Specifically, there were only two studies with three different protocols included in the analyses of PTH and 25OHD responses by Vitamin D combined with calcium supplementation. Another limitation was that a significant heterogeneity existed indicating that the studies might not have measured the same treatment effects, and a random effects model was used to account for the presence of heterogeneity. Furthermore, we did not use the PRISMA checklist for reporting the systematic reviews.

## 5. Conclusions

In conclusion, this meta-analysis identified that obese populations may respond to 1000 IU of vitamin D supplementation for PTH suppression. It also demonstrated that vitamin D supplementation alone can significantly suppress PTH, while raising 25OHD. The evident gap between the daily dose of vitamin D needed to achieve PTH suppression vs. the highest increment of 25OHD elevation should be thoroughly investigated in future studies including the role of different formulations of vitamin D and cholecalciferol absorption. The meta-analysis showed that lower doses of vitamin D along with calcium can also significantly suppress PTH. Future research is needed to determine if there is a dose variation among obese and overweight individuals and whether these reductions in serum PTH levels and increases in serum 25OHD following vitamin D supplementation are associated with clinically meaningful outcomes.

## Figures and Tables

**Table 1:** Overview of vitamin D supplementation trials in overweight and obese populations ^a^.

Study (Duration, M/F (*n*), Age Ethnicity)	Dose Vit. D (per Day)	Mean BMI (kg/m^2^)	Initial PTH (pg/mL)	Final PTH (pg/mL)	∆PTH (pg/mL)	Initial 25OHD (ng/mL)	Final 25OHD (ng/mL)	∆25OHD (pg/mL)
Zitterman et al. [16] (1 year) 62/138. 48.1 ± 10.2 years Not disclosed	3320 IU (*n* = 82)	33.7	44.7 ± 31.0	32.3 ± 19.3	−11.8 ± 20.8 *	12.0 ± 7.0	34.2 ± 23.0	22.2 ± 22.3 *
	Placebo (*n* = 83)	33.0	46.0 ± 7.0	37.5 ± 15.6	−8.7 ± 24.3 *	12.1 ± 8.0	16.8 ± 14.0	4.7 ± 14.5 *
Gaiser et al. [17] (12 weeks) 53/0. 28.7 ± 5.0 years Caucasian: 87%; Other: 13%	2000 IU(*n* = 17)	30.0	32.56 ± 11.7	26.6 ^b^	−6.0 ± 10.4	21.4 ± 6.7	26.6 ^b^	5.2 ± 5.6 *
	1000 IU (*n* = 20)	27.4	38.8 ± 20.3	39.4 ^b^	0.6 ± 16.5	21.0 ± 5.6	22.8 ^b^	1.8 ± 4.5 *
	Placebo (*n* = 16)	26.2	41.3 ± 21.8	37.2 ^b^	−4.1 ± 22.4	19.9 ± 7.0	21.2 ^b^	1.3 ± 5.2 *
Harris et al. [18] (12 weeks) 44/45. 56.6 ± 11.4 years African American	4000 IU (*n* = 46)	32.6	66.0 ± 27.9	48.6 ^b^	−17.4 ± 2.7 *	15.9 ± 5.2	32.4 ± 11.2	16.7 ± 1.2 *
	Placebo (*n* = 43)	31.9	64.5 ± 28.9	59.5 ^b^	−5.0 ± 2.6 *	15.3 ± 6.2	15.0 ± 6.4	−0.4 ± 1.2 *
Wood et al. [19] (1 year) 0/252. 63.8 ± 2.1 years Caucasian	1000 IU (*n* = 35)	<25.0	46.7 ± 10.5	40.4 ^b^	−5.6 ± 7.3 *	13.7 ± 5.9	33.0 ^b^	19.2 ± 8.5 *
	400 IU (*n* = 37)	<25.0	46.7 ± 10.5	42.3 ^b^	−3.7 ± 8.7 *	13.7 ± 5.9	27.0 ^b^	13.3 ± 9.3 *
	Placebo (*n* = 35)	<25.0	46.7 ± 10.5	43.3 ^b^	−2.7 ± 8.0 *	13.7 ± 5.9	14.4 ^b^	−0.7 ± 0.5 *
	1000 IU (*n* = 45)	25.0–29.9	49.5 ± 13.3	40.6 ^b^	−8.4 ± 10.6 *	13.6 ± 5.7	31.0 ^b^	15.5 ± 7.5 *
	400 IU (*n* = 44)	25.0–29.9	49.5 ± 13.3	43.6 ^b^	−5.4 ± 9.5 *	13.6 ± 5.7	26.9 ^b^	13.4 ± 7.9 *
	Placebo (*n* = 47)	25.0–29.9	49.5 ± 13.3	47.5 ^b^	−1.5 ± 7.3 *	13.6 ± 5.7	12.7 ^b^	−0.8 ± 4.7 *
	1000 IU (*n* = 16)	>30.0	51.4 ± 13.3	43.0 ^b^	−8.0 ± 7.6 *	13.0 ± 6.5	30.2 ^b^	17.2 ± 6.2 *
	400 IU (*n* = 16)	>30.0	51.4 ± 13.3	48.0 ^b^	−3.0 ± 5.4 *	13.0 ± 6.5	25.6 ^b^	12.6 ± 8.2 *
	Placebo (*n* = 18)	>30.0	51.4 ± 13.3	49.8 ^b^	−1.2 ± 5.2 *	13.0 ± 6.5	10.4 ^b^	−2.6 ± 5.2 *
Salehpour et al. [20] (12 weeks) 0/77. 37.5 ± 7.5 Not disclosed	1000 IU (*n* = 39)	30.1	13.3 ± 6.7	11.4 ± 4.8	−1.9 ± 4.8 *	14.7 ± 12.0	30.0 ± 8.8	15.3 ± 12.8 *
	Placebo (*n* = 38)	29.5	13.3 ± 6.7	16.2 ± 7.6	1.9 ± 4.8 *	18.8 ± 12.8	20.6 ± 12.4	1.8 ± 5.6 *
Kamycheva et al. [21] (1 year) 120/198. 49.2 ± 11.2 years Not disclosed	5714 IU (*n* = 107)	34.6	49.0 ± 14.9	39.4 ± 14.5	−9.2 ± 14.3 *	22.3 ± 6.2	46.3 ± 10.9	32.3 ± 12.4 *
	2857 IU (*n* = 103)	34.6	52.1 ± 16.6	44.4 ± 16.6	−7.7 ± 13.3 *	21.1 ± 7.4	36.1 ± 8.4	16.9 ± 8.6 *
	Placebo (*n* = 108)	34.6	53.7 ± 16.7	50.9 ± 17.9	−2.8 ± 14.3 *	21.3 ± 6.4	34.2 ± 13.7	12.9 ± 6.6 *
Drincic et al. [22] (21 weeks) 25/37. 45.8 ± 12.7 years Caucasians	1000 IU (*n* = 22)	36.7	22.3 ± 8.6	19.9 ^b^	−2.4 ± 4.8 *	20.3 ± 6.4	32.7	12.4 ± 9.7 *
	5000 IU (*n* = 20)	36.1	22.1 ± 13.0	21.3 ^b^	−0.8 ± 8.0	26.5 ± 6.7	54.3	27.8 ± 10.2 *
	10,000 IU (*n* = 20)	37.9	28.7 ± 15.6	23.8 ^b^	−4.9 ± 9.4 *	23.2 ± 15.2	73.9	50.7 ± 16.4 *
Wamberg et al. [23,24] (26 weeks) 15/37. 40.4 ± 7.4 years Not Disclosed	7000 (*n* = 22)	36.1	50.5 ± 2.3	42.9 ± 14.3	−7.6 *^,b^	13.8 ± 4.0	44.0 ± 6.9	30.8 *
	Placebo (*n* = 21)	35.0	ND	ND	ND	13.8 ± 4.0	18.7 ± 8.5	−5.2 *
Jorde et al. [25] (1 year) 159/282 47.0 ^b^ years Not disclosed	2857 IU + 500 mg Ca^2+^ (*n* = 116)	33.3	47.6 (27.6–131.4)	41.9 (19.0–121.9)	−5.7 *^,b^	20.9 (6.2–44.6)	35.1 ^b^	14.2 ^b^
	5714 IU + 500 mg Ca^2+^ (*n* = 106)	33.5	45.7 (21.9–104.7)	45.7 (21.9–94.3)	−7.6 *^,b^	22.1 (6.7–38.8)	44.9 (18.7–77.4)	22.8 ^b^
	Placebo (*n* = 112)	43.8	50.5 (21.9–104.7)	50.5 (21.9–104.7)	−1.0 *^,b^	21.0 (7.4–39.8)	20.0 (8.1–39.9)	−1.0 ^b^
Pilz et al. [26] (1 year) 54/0. 48.3 ± 8.2 years Not disclosed	3332 IU (*n* = 31)	33.1	39.4 ± 18.6	33.7 ± 18.9	−5.7 ± 15.9	13.0 ± 8.0	34.6 ± 27.5	21.4 ± 26.1 *
	Placebo (*n* = 23)	32.5	48.3 ± 34.6	39.3 ± 14.1	−9.0 ± 29.4	11.9 ± 9.5	14.2 ± 3.2	34.6 ± 27.5 *
Pittas et al. [27] (3 years) 181/219. 71.2 ± 0.5 years Caucasian	700 IU + 500 mg Ca^2+^ (*n* = 108) ^NFPG^	26.1	36.1 ± 1.9	31.6 ^b^	−4.5 ± 1.3 *	32.6 ± 1.5	44.4 ^b^	11.8 ± 1.4 *
	Placebo (*n* = 114) ^NFPG^	26.2	40.0 ± 1.9	46.3 ^b^	6.3 ± 1.1 *	28.2 ± 1.1	27.9 ^b^	−0.34 ± 0.9 *
	700 IU + 500 mg Ca^2+^ (*n* = 45) ^IFPG^	27.8	42.9 ± 2.9	34.9 ^b^	−8.0 ± 2.4 *	28.5 ± 2.1	41.0 ^b^	12.5 ± 1.8 *
	Placebo (*n* = 47) ^IFPG^	28.1	38.1 ± 1.9	42.7 ^b^	4.6 ± 1.5 *	32.5 ± 1.9	29.4 ^b^	−3.1 ± 1.2 *
Harinarayan et al. [28] (12 weeks) 15/21. 31.0 ± 5.8 years Not disclosed	9571 IU + 1000 mg Ca^2+^ (*n* = 36)	34.3	57.9 ± 29.3	36.3 ± 22.5	−21.6 ± 22.5 *	8.8 ± 4.5	36.8 ± 18.7	28.1 ± 18.3 *
	No placebo	ND	ND	ND	ND	ND	ND	ND
Martins et al. [29] (12 weeks) 78/52. 18 to 70 years African American	3333 IU (*n* = 65)	>25.0	43.4 ± 19.9	37.5 ± 16.2	−5.9 *^,b^	17.0 ± 5.2	34.5 ± 7.1	7.0 ^b^
	Placebo (*n* = 65)	>25.0	49.9 ± 33.6	49.7 ± 37.6	−0.2 *^,b^	16.5 ± 5.0	17.2 ± 6.4	0.7 ^b^
Talwar et al. [30] (3 years) ^c^ 0/208. 60.6 ± 6.2 years African American	800 IU/2000 IU (*n* = 104) (3 months)	29.0	44.2 ± 19.3	33.0 ± 14.4	11.2 *^,b^	18.8 ± 6.7	28.6 ± 8.2	9.8 ^b^
	Placebo (*n* = 104) (3 months)	30.0	42.4 ± 18.4	34.4 ± 15.5	−8.0 *^,b^	17.3 ± 8.2	15.6 ± 6.7	−1.7 ^b^
	800 IU/2000 IU (*n* = 104) (24 months)	29.0	44.2 ± 19.3	39.3 ± 17.7	−4.9 *^,b^	18.8 ± 6.7	26.4 ± 8.6	7.6 ^b^
	Placebo (*n* = 104) (24 months)	30.0	42.4 ± 18.4	38.2 ± 15.3	−4.2 *^,b^	17.3 ± 8.2	16.6 ± 7.3	−0.7 ^b^
	800 IU/2000 IU (*n* = 104) (27 months)	29.0	44.2 ± 19.3	36.3 ± 15.9	−7.9 ^b^	18.8 ± 6.7	34.9 ± 9.0	16.1 ^b^
	Placebo (*n* = 104) (27 months)	30.0	42.4 ± 18.4	35.5 ± 15.0	−6.9 ^b^	17.3 ± 8.2	18.1 ± 7.2	0.8 ^b^
Sneye et al. [31] (1 year) 159/286. 47.6 ± 11.4 years Not disclosed	5714 IU + 500 mg Ca^2+^ (*n* = 116)	35.0	49.5 ± 15.0	40.8 ^b^	−8.7 ± 14.4 *	21.8 ± 6.7	45.7 ^b^	23.9 ± 8.4 *
	2857 IU + 500 mg Ca^2+^ (*n* = 106)	34.4	51.0 ± 17.0	43.4 ^b^	−7.6 ± 13.7 *	20.6 ± 7.4	35.7 ^b^	15.1 ± 6.3 *
	Placebo + 500 mg Ca^2+^ (*n* = 112)	35.1	53.8 ± 20.9	51.3 ^b^	−2.5 ± 15.1 *	21.3 ± 6.2	20.4 ^b^	−0.9 ± 3.8 *
Gannage-Yared et al. [32] (12 weeks) Not disclosed	800 IU + 1000 mg Ca^2+^ (*n* = 47)	28.1	48.6 ± 18.6	42.2 ± 16.4	−6.36 ^b^	10.6 ± 6.6	25.8 ± 6.6	15.3 ^b^
	No placebo	ND	ND	ND	ND	ND	ND	ND
Carillo et al. [33] (12 weeks) Not disclosed 26.1 ± 4.8 years Not disclosed	4000 IU + 500 mg Ca^2+^ (*n* = 10)	30.6	36.1 ± 10.7	24.0 ± 12.0	−11.7 ± 10.6 *	20.8 ± 8.3	33.4 ± 7.2	12.3 ± 8.4 *
	Placebo + 500 mg Ca^2+^ (*n* = 13)	31.9	43.2 ± 34.6	43.5 ± 29.8	0.3 ± 8.9 *	18.1 ± 6.5	23.5 ± 6.0	5.5 ± 7.9 *

Boxes indicate trials included in meta-analysis. Initial and final PTH and 25OHD values are reported in either mean ± SD or median (25%, 75%). ND = no data; * = results significant; a = Mean and SD are reported in the tables, b = Standard deviation or confidence interval not reported; c = 800 IU/day vitamin D3 for the first 2 years, 2000 IU/day vitamin D3 for the last year of this study; NFPG = normal fasting plasma glucose; IFPG = impaired fasting plasma glucose.
